# Effectiveness of a nutritional educational program for weight gain management among medical students in the faculty of medicine at Ain Shams University: an application of the health belief model

**DOI:** 10.1186/s41043-025-00803-8

**Published:** 2025-04-02

**Authors:** Nourhan B. Abd El Samad, Eman A. Ghanem, Sahar A. Dewedar, Azza M. Hassan

**Affiliations:** Department of Community, Environmental and Occupational Medicine, Faculty of Medicine, Ain Shams University, Cairo, 1181 Egypt

**Keywords:** Health belief model intervention, Medical education, Overweight and obesity, Weight management

## Abstract

**Background:**

Obesity and overweight are widespread among individuals in both developed and emerging nations. Obesity is a global health issue, and its prevalence has been increasing in Egypt for several decades. The health belief model (HBM) is a comprehensive framework model that has an important role in preventing disease and promoting health.

**Objectives:**

This study aims to measure the prevalence of overweight and obesity among medical students in the faculty of medicine at Ain Shams University and to determine the effect of nutritional educational program on body mass index and health belief model scales among them.

**Methods:**

This study was conducted in the Faculty of Medicine at Ain Shams University among medical students in 2 phases: phase 1, a descriptive cross-sectional study to calculate the prevalence of the disease; and phase 2, an intervention study that included a sample of 100 medical students whose body mass index was greater than or equal to 25. The study tool is a self-administered questionnaire based on the health belief model for weight control behavior that is tested by the participants before and after the intervention program.

**Results:**

Most participants had a normal BMI, while 40% were overweight or obese. There was a statistically significant difference in the HBM scores before and after the intervention sessions. The mean BMI before the intervention was 29 ± 0.25 and had improved after the intervention session to 29, where there was a highly statistically significant difference (*p*-value < 0. 001).

**Conclusions:**

Nutritional education sessions based on the health belief model help in improving the knowledge and habits of high-risk medical students for weight gain. There were substantial changes between before and after the nutrition program interventions.

**Supplementary Information:**

The online version contains supplementary material available at 10.1186/s41043-025-00803-8.

## Introduction

The epidemic of obesity poses serious risks to public health because of its contribution to the occurrence of various chronic diseases, such as fatty liver, type two diabetes mellitus, cardiovascular diseases, cerebrovascular diseases, musculoskeletal disorders, Alzheimer’s disease, and certain types of malignancies, that lead to deterioration in health outcomes and mortality [1]. The World Obesity Federation now considers obesity a disease that progresses over time rather than merely a risk factor for other conditions [2]

There has been a global surge in obesity. According to estimations from the World Health Organization (WHO), the prevalence of adult obesity worldwide has doubled since 1990. In 2022, 43% of adults aged 18 years and over were overweight, and 16% were obese [3].

A stepwise survey conducted in Egypt in 2017, revealed that about 63% of people were overweight and 36% were obese, with a higher obesity rate in females [4]. Comparing data from 2012 to 2017 showed an increase in overweight from 62 to 63% and in obesity from 31. 1% to 35. 7% [5]. In 2018, Egypt launched a large screening campaign for hepatitis C, measuring weight and height. Obesity prevalence was noted at 39. 84%, with females showing higher rates. Egypt is ranked fourth globally in obesity and first for female obesity [6].

The HBM, which is a psychosocial model used in health promotion and disease prevention interventions, is composed of six constructs that affect the decision to practice a certain health-related behavior as follows: perceived susceptibility to the health condition; (perceived severity reflects the awareness of the effect of disease on health; perceived benefits reflect the benefits of practicing the required behavior; perceived barriers and costs; and cues to action, either internal cues or external cues; and perceived self-efficacy, which reflects one’s ability to carry out an action [7].

The transtheoretical model (TTM) provides a framework for improving the efficacy of weight control interventions. This model focuses on assessing an individual's willingness to alter their behaviors, assuming that behavioral change is complex, unfolding in a sequence of stages [8]. The model comprises S change phases and P procedures phases. The five stages of the S-change phase include pre-contemplation (PC), contemplation (C), preparation (Prep), action (A), and maintenance [9].

The prevention of weight gain is thought to be easier than weight loss; therefore, weight gain is considered the cornerstone of primary weight management [10]. Individuals, families, health care, and society all share responsibility. However, implementing effective evidence-based interventions that target weight management is challenging [11]. Studies have shown that factors such as perceived severity, benefits, self-efficacy, and cues to action significantly predict behavioral intention for weight management [12].

Our future medical professionals are our students, who are exposed to sedentary lifestyles, stress, disordered eating habits, and excessive use of technology, which are common causes of obesity [13]. Therefore, developing effective weight management strategies is considered a path to promote weight loss.

Nutrition education includes several actions, such as assessing information, expanding awareness of the importance of diet and healthy behaviors, assisting in developing participants' abilities, and inspiring them to adapt to healthy dietary habits [14].

Owning to the importance of education in promoting preventive behaviors such as combating overweight and obesity, the current study was carried out to evaluate the impact of a nutrition education intervention and investigate the effect of educational programs based on health belief models on promoting behaviors to prevent weight gain among medical students in the faculty of medicine at Ain Shams University.

The purpose of this study was to measure the prevalence of overweight and obesity among medical students in the faculty of medicine at Ain Shams University and to evaluate the effect of a nutrition educational program based on the health belief model on these conditions.

## Subjects and methods

### Research design

The study was carried out in two phases:

- Phase I: cross-sectional study
- Phase II: Intervention quasi-experimental design where a pre-posttest was utilized in this study.

### Study setting

This study was carried out in the faculty of medicine at Ain Shams University.

### Subjects and sampling techniques

To measure the prevalence of overweight/obesity, the study was carried out on a convenience sample of 800 medical students, aged more than 18 years who, from all grades and 100 overweight/obese students, were needed to detect expected improvements in HBM scores after the intervention according to the following criteria:

- Body mass index BMI ≥ 25.0 kg/m2
- Free from any chronic disease.

### Study tools

For data collection in the two phases, two tools were utilized as follows:

- First tool for Phase 1: Measurements for weight, height, and BMI and data for gender and academic grade were collected.
- The second tool for Phase 2: was divided into two sections and included self-administered pre-posttest questionnaires based on the health belief model for weight-management behavior:

#### Section 1

Sociodemographic data (for example, age, gender, academic grade, BMI).

#### Section 2

This section is based on HBM variables (perceived severity, perceived susceptibility, perceived benefit, perceived barrier, cues to action, and self-efficacy), the behavioral intention of the weight management scale, and the S change questionnaire for stages of change model (Andrés A et al., 2011) [15]. The questionnaire used to measure the HBM variables was obtained from (Park DY, (2011) [16] and (McArthur et al., (2017) [17].

### Scoring system for the HBM

The overall scale was measured via a five-point Likert-type scale dispersed across the HBM subscales to evaluate changes in students' HBM toward weight gain management. The participants can rank their level of agreement or disagreement with the statements from (1) strongly disagree to (5) strongly agree.

### Content validity and reliability

The content validity index (CVI) and content validity ratio (CVR) indicated satisfactory results for each item (CVI range: 0.78–1.00; CVR range: 0.80–1.00) [18].

Cronbach’s alpha is used to calculate reliability. Alpha coefficients equal to or greater than 0.70 are considered satisfactory (Cronbach LJ, 1951) [19]. The overall reliability of the tool based on Cronbach’s alpha was 0.92. The intraclass correlation coefficient showed excellent agreement (ICC = 0.86) [18].

### Study procedures and framework study design

The study involved several stages, such as the preparation, intervention, and evaluation stages.

#### Preparatory phase

The program included the distribution of self-administered questionnaires based on the HBM among medical students for weight management.

#### Intervention phase

Medical students' needs, interests, understanding, and knowledge levels were taken into consideration when nutritional education sessions, which were based on the HBM, were designed. The intervention aimed to improve students’ nutritional awareness and habits.

The intervention was strengthened by a colorful educational booklet and posters. Each participant received a colorful educational booklet as a gift to attract them.

#### Sessions

**Fig. 1:**
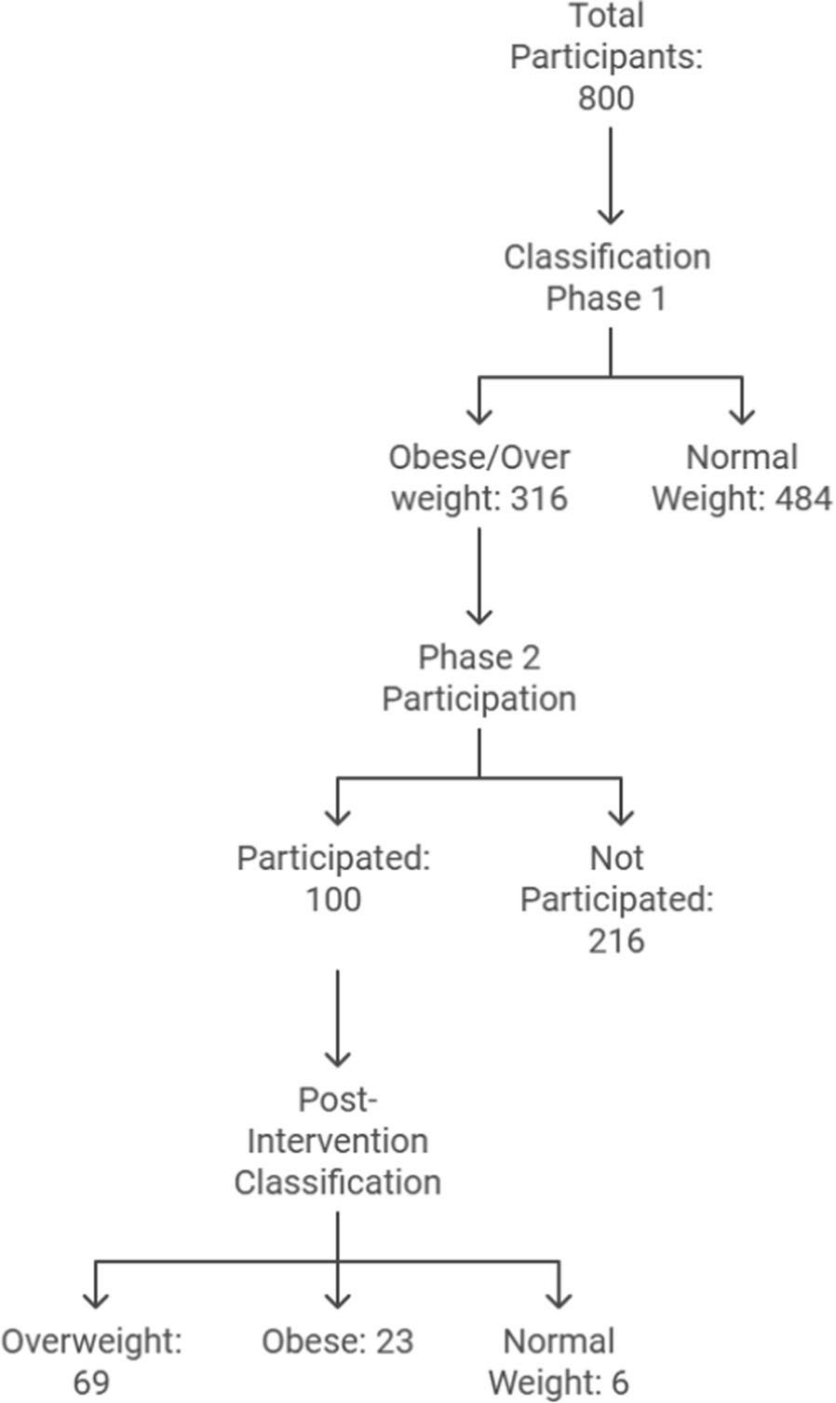
A study design for the participants over the two phases

**Fig. 2:**
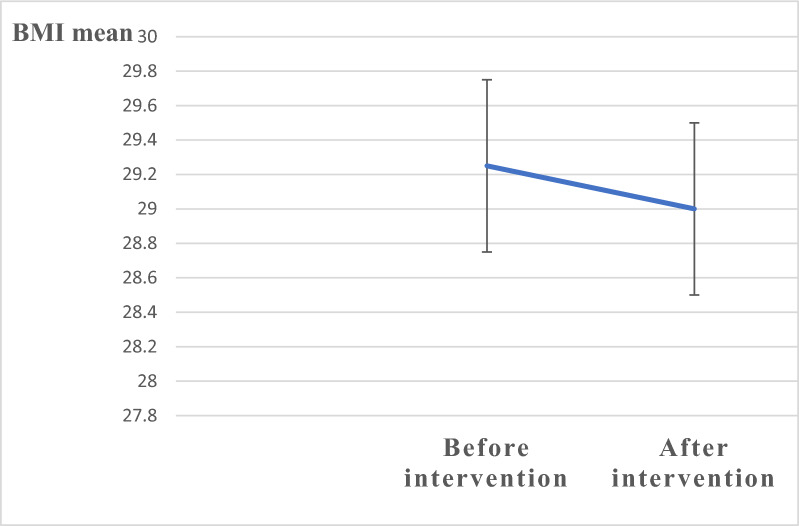
Error bar graph showing improvement in body mass index: before and after intervention

The sessions took place in available classes chosen for the implementation of the HBM model in the faculty. The sessions were classified into; one dedicated to the theoretical part and the other to the practical application. Each session took about 90 min integrated with teaching points and before going on to a new topic; questions were asked to check the participants’ recall and understanding of the material already covered (Figs. 1, 2).

An overview of the program and its goals was provided at the beginning of the initial session. From the second session onward, each session began with a summary of the previous sessions.

The theoretical part of the sessions includes:

- Principles of proper nutrition
- Define obesity.
- Risk factors for obesity.
- Complications of obesity
- Methods used for weight reduction.
- HBM constructs related to factors affecting weight management
- Identify types of physical exercise.
- Explain the components of a balanced diet.
- Healthy lifestyle and Maintenance of weight management
- Explain the importance of BMI measurements.
- The practical part includes:
- Demonstrate preparation of healthy food
- Calculating the dietary calories
- Reading the nutrition value labels
- Demonstrate Types of physical exercises.

### Evaluation phase

This phase aimed to assess the level of change in medical students' perceptions and lifestyles to avoid the implications of health problems through the program's implementation. This was accomplished by measuring BMI and administering a posttest resembling the pretest to the study participants three months after the program's completion to estimate the effect of the program on medical students' obesity-related beliefs and practices, as well as the effect of using the health belief model to improve their health status and healthy practices.

### Statistical analysis

Data were revised, coded, analyzed, and tabulated using the percentage and number distributions for qualitative variables, however using mean and standard deviation for quantitative variables.

Using SPSS version 27, suitable statistical tests such as paired t-test, chi-square, and independent T-test were performed. A Comparison between qualitative variables was made using Pearson’s Chi-square to compare BMI classifications related to gender. A paired t-test was used for between-group comparisons of continuous variables with normal distributions. T-tests compared means of two groups of continuous variables regarding the scores on the HBM subscales based on demographics and BMI change scores.

-Statistically significant differences at *p*-values less than or equal to 0.05.

#### Ethical consideration

Ethical and administrative approvals from the Ain Sham Faculty of Medicine Ethical Committee were obtained, (under the number code FAMSU MD 125/2022 (FWA 000017585) 1/6/2022))

Informed verbal consent was obtained from the participants.

The confidentiality and privacy of the data were assured.

## Results

The baseline characteristics of the medical students are displayed in Table 1, which clearly shows that the participants’ mean age was 21 years; where 56% were males and 44% were females had been participated.

**Table 1:** Baseline characteristics of the study participants in phase 1 (n = 800)

Variable		N	(%)
Age	Mean ± SD = 21± 1.4 Range = 19 – 23		
Sex	Male	447	55.8
	Female	353	44.2
Academic Year	Year1 Year2 Year3 Year4 Year5	160 160 160 160 160	20 20 20 20 20
BMI category	Underweight Normal Overweight and Obese	16 468 316	2 58.5 39.5

The relationship between BMI category and gender is illustrated in Table 2, which shows that the BMI of most males 67% was normal and approximately 31% were overweight or obese; whereas 50% of females were overweight or obese and approximately 48% were normal in weight according to the BMI categories. There was a statistically significant difference (*p* < 0.001) between the BMI categories between the groups.

**Table 2:** Body mass index classification related to gender among the study participants in phase 1 (n = (800)

Gender	Underweight (n = 16)		Normal (n = 468)				Overweight and obese (n = 316)			Total		X2	*P* value
	N	(%)	N	(%)			N	(%)		N	(%)		
Male (447)	9	2	299		67		139		31	447	100	30.304	< 0.001*
Female (353)	7	2	169		48		177		50	353	100		
Total	16	2	468			58.5	316		39.5	800	100		

X2 = Chi square test

*P value ≤ 0.05 is considered statistically significant and a chi-square test was used

The intervention phase: included the baseline characteristics of the intervention phase, in which approximately 100 students had participated; most participants were in the fourth academic year. Among the males who participated, 69% were overweight and 31% were obese, among the females who participated, 72% were overweight and 28% were obese. Concerning participants’ readiness for weight management, except for a small number of participants in the action and maintenance stage, the majority were in the contemplation stage, followed by the preparation stage, as shown in Table 3.

**Table 3:** Baseline characteristics of the intervention phase among the study participants (n = 100)

Variable		N	(%)
Age	Mean ± SD = 21± 1.3 Range = 19 – 23		
Sex	Male	32	32
	Female	68	68
Academic year	Year1 Year2 Year3 Year4 Year5	12 18 20 34 16	12 18 20 34 16
BMI category	Overweight Obese	71 29	71 29
Male* n=(32) Female** (n=68)	Overweight Obese Overweight Obese	22 10 49 19	69 31 72 28
Transtheoretical model stages	Precontemplation Contemplation Preparation Action Maintenance	0 83 12 4 1	0 83 12 4 1

BMI, body mass index

*n = 32

**n = 68

Differences in the scales of the HBM before and after the nutrition education session among the participants are illustrated in Table 4. The intervention resulted in statistically significant improvements across all the scales measured via the HBM.

**Table 4:** Comparison of health belief model constructs related to weight gain management before and after the intervention nutrition education program

Items	Pre-education (Mean ± S.D)	Post-education (Mean ± S.D)	Paired t-test	*P* value
Perceived Severity	43 ± 2.9	48 ± 3.8	11.7	< 0.001*
Perceived susceptibility	28.7 ± 1.7	30 ± 2.5	4.7	< 0.001*
Perceived barrier	49 ± 4.5	25 ± 2.5	−46.6	< 0.001*
Perceived benefit	48 ± 3	52 ± 5	6.45	< 0.001*
Perceived cues to action	42 ± 3	48 ± 3.9	13.6	< 0.001*
Perceived self-efficacy in diet	47 ± 5	69.6 ± 4	32.6	< 0.001*
Perceived self-efficacy in exercise	17 ± 2	23 ± 1.7	23.2	< 0.001*
Perceived total efficacy	64 ± 6	93 ± 5	36.6	< 0.001*
The behavioral intention of weight management	18 ± 1.9	21 ± 1.5	16	< 0.001*
Total all	275 ± 11	297 ± 10	19.8	< 0.001*

*P value ≤ 0.05 is considered statistically significant. A paired t-test was used

The differences in BMI before and after the nutrition intervention sessions revealed that participants benefitted from reducing BMI after attending the intervention sessions, as revealed in Table 5.

**Table 5:** Comparison of BMI before and after the intervention nutrition education program

	Pre-education (Mean ± S.D)	Post education (Mean ± S.D)	Paired t-test	*P* value
BMI	29.25 ± 3	29 ± 3	−14.3	< 0.001***

BMI, body mass index

*P value ≤ 0.05 is considered statistically significant. A paired t-test was used

The relation between gender and changes in BMI and HBM subscales scores before and after the intervention are illustrated in Table 6, where the findings showed a significant gender difference in BMI changes where mean values of males (0.378 ± 0.248) greater than females(0.26 ± 1.77), suggesting males experienced greater changes and significant difference regarding the HBM scores where females showed greater improvement (23 ± 11) compared to males (17 ± 8.7) after the intervention.

**Table 6:** Relation between gender and change in the BMI and HBM scores before and after the intervention

	Gender		T-test	*P* value
Items	Male n = 32	Female n = 68		
BMI change	0.378 ± 0.248	0.26 ± 1.77	2.715	0.008*
Change in the pre and post-HBM scores	17 ± 8.7	23 ± 11	−2.6	0.010*

*P value ≤ 0.05 is considered statistically significant. An Independent t-test was used

## Discussion

By utilizing the health belief model (HBM) as a conceptual model, the goal of this research was to determine the variables affecting the behavioral intentions of students to control their weight gain.

The current study revealed that overweight and obesity are highly common among medical students. Therefore, programs based on behavior medicine could help students avoid unhealthy weight gain or assist them in losing weight by providing a healthy weight-control strategy that includes access to wholesome food options on campus, increased opportunities for physical activity, and knowledge about better lifestyles.

According to the findings of the present study, the majority of participants were 21 years of age, most of them were males and were more likely to cooperate to participate in the first phase, where weight, height, and body mass index measurements were taken. The majority of participants were in the normal category according to the WHO BMI classification, followed by the overweight and obese class, which reflects the increase in the burden of overweight and obesity in the future, where it constituted about 40% of the studied sample.

Similarly, among undergraduate nursing students at Mansoura University, 48.75% were class I obese, 32.5% were class II obese, 12.5% were class III obese, and only 6.25% were overweight [20].

Additionally, a study conducted among college students in Iran; reported that the average age of their student participants was 22 and that approximately 70% were normal in weight [19].

Concerning phase 1 in the current study, a highly significant relationship (*p* =  < 0.001) was found between the BMI categories and the sex of the participants.

Compared with the percentage of male students in the same category, the percentage of female students classified as overweight or obese was significantly greater. The percentage of males who were normal was greater than that of females. This explains why the majority of overweight or obese individuals are females, which suggests that BMI is variable across genders, and more interventions are needed, especially for females, to reduce the negative consequences of obesity.

The findings of the present study agreed with those of a study conducted among university students at Jazan University, Saudi Arabia, which revealed a statistically significant difference between the BMI categories and the gender of the students, whereas the findings contradict this of Age, which did not show any significant associations (*p* > 0.05) with BMI categories [21].

The current findings correlate with those of a study conducted among Malaysian adults, which revealed a highly significant difference between participants’ age and sex and their BMI categories; their data showed that obesity was greater among females than among males, whereas the percentage of individuals with a normal BMI was greater among males [22].

The transtheoretical model (TTM) was used to assess a person's readiness to change. The results of the present study showed that most of the participants were in the contemplation stage, where they had the intention for weight control and management. This is in line with a study conducted in Iran among 250 overweight and obese adolescents, which revealed that the majority of participants were in the contemplation stage (56.8%), followed by pre-contemplation (23.6%), preparation (7.6%), action (5.2%) and maintenance (6.8%), where 80% of those seeking weight control, were in the inactive stages (pre-contemplation, contemplation, and preparation), with only 20% in the active stages (action and maintenance) [9].

Moreover, regarding the HBM scales, the current study revealed a significant difference between undergraduate medical students’ perceptions of susceptibility and external cues to action in terms of their BMI categories, either overweight or obese. Individuals with obesity in the intervention group commonly exhibit a diminished perception of severity, susceptibility, obstacles, and benefits with those who are overweight, matching findings from a similar study that suggest that individuals with obesity frequently regard their weight status as less severe [19].

The intervention phase aimed to determine whether the educational nutrition program was effective in helping study group participants maintain a healthy weight. The ‘‘behavior modification’’ interventional program (including nutritional education, modifying, and changing dietary habits, teaching exercise programs and their benefits to the students, and teaching nutritional facts to the students) is effective in reducing the BMI among students.

In this study, the nutrition education sessions had an impact on the perceived HBM constructs among the participants where all the constructs had been changed and improved after the intervention sessions. The mean scores of all HBM constructs were significantly higher after the intervention than those before the intervention. Nevertheless, the mean score of perceived barriers decreased significantly after the intervention, which could be justified on the grounds that there is an inverse relationship between the scores of perceived benefits and barriers and related to the background of the participants as they were medical students and aware by the benefits of weight control.

The perceived barrier showed a relative change where most of the participants before the intervention had a misconception about healthy nutrition where they thought it was expensive, tasteless, not available anywhere, and took a long time to prepare and purchase because the participants assumed that numerous obstacles prevented them from practicing healthy behaviors to maintain weight and that all these concepts had changed and corrected after the intervention.

The perceived benefit increased even though the participants were medical students and well-known about the severity and benefits, but they had inadequate nutrition knowledge and missed more information that improved after the intervention.

The intervention altered the perceived health belief model (HBM) scales, particularly about the perception of susceptibility to being overweight or obese, which is now recognized as a disease. Participants became more aware of their vulnerability to obesity when engaging in unhealthy behaviors. Additionally, there was a notable increase in self-efficacy, particularly concerning dietary habits. Participants demonstrated a greater awareness of their food choices, portion sizes, and the quality of the food they consumed. Furthermore, they exhibited increased confidence in their ability to engage in physical activity, understanding that even moderate exercise, such as walking for approximately 15 min a day, two to three times a week, is beneficial. They recognized that it is not necessary to invest excessive time or engage in vigorous activities to achieve meaningful lifestyle changes.

There is in co-ordinance with a study conducted at the University of Mashhad Medical Sciences in Iran; it was found that the mean scores before and one month after the educational intervention in all constructs of the HBM and the range of high-risk behaviors (other than smoking behavior) in the experimental group compared to the control group was statistically significant (*p* < 0.001) [23].

These previous findings are in the same line with a study conducted in Iran who studied "factors influencing weight management behavior among college students: An application of the HBM in Tabriz, Iran" and found that after application of HBM, the mean scores of the items perceived severity, susceptibility, perceived barriers to adopting healthy eating and physical activity habits, perceived barrier, perceived benefits to adopting healthy eating and physical activity habits, perceived cues to action for weight management, self-efficacy in dieting, and behavioral intention of weight management showed significant differences (*P* ≤ 0.001) [19].

Additionally, a similar study carried out on female secondary school students at El-Sharabia District in North Cairo; revealed significant differences (*P* ≤ 0.001) in the total HBM subscales before and after the intervention regarding perceived severity, perceived susceptibility, perceived barriers, perceived benefits, perceived self-efficacy, cues to action and behavioral intentions of weight management among the studied sample [24]^.^

There’s an agreement with a study carried out on female secondary governmental schools in the Zagazig district at Sharkia Governorate, Egypt that showed an improvement in the HBM constructs (perceived susceptibility, severity, benefits, barriers, self-efficacy, and cues to action) and a decrease in perceived barriers after Nutrition education program provided [25].

Similarly, in a study conducted among medical students from the Military University of Tehran in Iran, it was found that after the educational intervention was held; the mean scores of HBM constructs (perceived benefits, perceived barriers, perceived threat, and self-efficacy) considerably improved; which indicate the show that the intervention was successful and had a beneficial impact [26].

The findings of the present study revealed a statistically significant difference between BMI scores before and after the intervention, where the mean BMI after the intervention was lower than that before the intervention at *p* < 0.001, which reflects that the students had acquired the correct knowledge and made behavioral modifications to help them avoid obesity and clarify the effective role of nutritional education in increasing awareness which helped the participants lose weight and face their barriers.

Our findings suggest that the HBM is a valuable tool for healthcare providers and can be used as a theoretical foundation for assessing preventative initiatives in obesity reduction and healthy lifestyle promotion.

These findings go with a study conducted among employees at the University of Mosul in Iraq; where there was a significant difference in BMI pre and post-test; This means that the intervention had a positive impact on the BMI [27]. Moreover, a study conducted in junior high school in the center of Thailand showed a decrease in BMI among students who participated in the intervention group [28]. All these changes suggest the valuable role of the HBM-based intervention program as it explains that students' beliefs about health problems and the perceived benefits, barriers to change their behavior and self-efficacy explain the students’ engagement in health-promoting behavior because HBM scales serve as a stimulus for the students to change and maintain health-promoting behavior.

Interestingly, the findings of the present study matched with those of a study performed in Iran that revealed an improvement in BMI among participants following the educational intervention, indicating the beneficial impact of model-based education in enhancing obesity-related lifestyles among adults [29].

### Limitations of this study

The findings from this study should be interpreted in light of several limitations. The study involved an intervention program characterized by its relatively intensive nature and constrained by a specific time frame, which may have resulted in insufficient long-term follow-up for the participants. Furthermore, the absence of a control group presents a limitation. Nevertheless, our objective was to evaluate an intervention that was practical and, therefore, likely to have broad applicability. The limited number of participants in the intervention phase can be attributed to their willingness to engage, as the intervention sessions required a significant commitment of their time, which was already occupied with lectures and practical components of their curriculum.

## Conclusions

In conclusion, the findings of the present study indicate an improvement in the HBM scores concerning overweight and obesity after the nutritional intervention sessions. The study also revealed participants’ positive behavioral intention toward weight management and their readiness for change to weight control in a better healthier life. The nutrition intervention program improved the BMI of the participants. This research may serve as a valuable resource for researchers in various countries, enabling its application among college students and other demographic groups to derive benefits from its findings.

### Recommendation

An educational program for healthy behaviors connected to dietary patterns, exercise, and daily physical activities should be implemented to reduce the inclination toward overweight and obesity among the students and to incorporate the nutrition curriculum throughout their academic years, even within the school years, to help raise awareness among the students toward a healthy diet.

## Supplementary Information

Supplementary Material 1

## Data Availability

The datasets used and/or analyzed during the current study are available from the corresponding author upon reasonable request.

## Abbreviations

A: Action
BMI: Body mass index
C: Contemplation
CVI: Content validity index
CVR: Content validity ratio
HBM: Health belief model
ICC: Intraclass correlation
PC: Precontemplation
P: Preparation
SD: Standard deviation
SPSS: Statistical package for social sciences
TTM: Transtheoretical model
USA: United States of America
WHO: World Health Organization
