# Supplementary material for: Effectiveness of a nutritional educational program for weight gain management among medical students in the faculty of medicine at Ain Shams University: an application of the health belief model
Source: J Health Popul Nutr. 2025 Apr 2;44:101. doi: 10.1186/s41043-025-00803-8 (PMC11967057; doi:10.1186/s41043-025-00803-8)
Supplement: Supplementary file 1 — Supplementary Material 1 [file 41043_2025_803_MOESM1_ESM.pdf]

Questionnaire in English

Health Belief Model questionnaire for weight management behaviour						
**Perceived severity**		Completely disagree	Disagree	Undecided	Agree	Completely agree
Being overweight could …	***Emotional/mental health subscale***					
	1. Make me feel anxious and stressed					
	2. Cause others to find me less physically attractive.					
	3. Make me unhappy and depressed.					
	4. Lower my self-esteem.					
	Physical health/fitness subscale					
	5. Makes it harder to do the physical activity or sports that I enjoy.					
	6. Makes it harder to get enough sleep.					
	7. Cause pain in my knees.					
	8. Have an adverse effect on my health in years to come.					
	9. increases my risk for diabetes, high blood pressure, cancer and other illnesses.					
	Social/professional subscale					
	10. Make it harder to make friends.					
	11. Makes it harder to get a job because of a lack of fitness.					
	12.Take fun out of socializing with friends.					
	13.Makes me unable to wear clothes I want.					
**Perceived susceptibility**		Completely disagree	Disagree	Undecided	Agree	Completely agree
I could become susceptible to being overweight if…	***Lifestyle subscale***					
	1. I get <30 min of moderate-intensity physical activity on most days					
	2. I consume sugary beverages, foods, or snacks daily or on most days.					
	3. I eat fried foods or snacks daily or on most days.					
	4. I eat at fast-food restaurants ≥3 times/wk.					
	5. I don't pay attention to the amounts I eat or drink.					
	Environmental subscale					
	6. One or both of my parents is overweight or obese.					
	7. I have a genetic history of being overweight or obese.					
**Perceived barriers**		Completely disagree	Disagree	Undecided	Agree	Completely agree
Practical concerns subscale						
1. Lower-calorie beverages, foods, and snacks are too expensive						
2. Grocery shopping and preparing healthy foods would take up too much of my time.						
3. Doing exercise/physical activity on most days would take up too much of my time.						
4. My job/studying means more to me than adopting healthy eating and physical activity habits						
Emotional/mental health subscale						
5. I do not have any motivation to adopt healthy eating and physical activity habits						
6. I enjoy eating fried foods and snacks more than baked, grilled or steamed versions						

7. I enjoy consuming sugary beverages, foods, and snacks more than lower-calorie versions					
8. I often turn to food when I want to feel comforted					
Awareness subscale					
9. I don’t know where to find accurate information about achieving and maintaining a healthy weight					
10. I don’t know how to plan physical activity into my daily schedule					
11. I don’t know where to shop for healthy beverages, foods, or snacks.					
12. I don’t know how to prepare low-calorie beverages, foods, or snacks.					
13. I don’t know how to choose low-calorie beverages, foods, or snacks.					

**Perceived benefits**		Completely disagree	Disagree	Undecided	Agree	Completely agree
It would benefit me to adopt healthy eating and physical activity habits by…	*Emotional/mental health subscale*					
	1. Reducing depression, anxiety and stress.					
	2. Helping me to improve my body image.					
	3. Improve my self-esteem					
	4. Improve my mood					
	Physical health/fitness subscale					
	5. Make it easier to do the exercise/sports I enjoy					
	6. Make me feel more energetic					
	7. Increase my chances of having good health now and in the future					
	8. Improve a symptom or health problem I have now					
	9. Help me become more physically fit to improve my job performance					
	10. Help me sleep better					
	11. Make it easier to accomplish my daily activities					
	Social/professional subscale					
	12. Providing a better marriage opportunity.					
	13. Make me feel more comfortable around others					

**Cue to action**		Completely disagree	Disagree	Undecided	Agree	Completely agree
I would adopt healthy eating and physical activity habits if…	*Internal cues*					
	1. I looked in the mirror and was dissatisfied with my body					
	2. My clothes fit uncomfortably tight					
	3. I developed a health problem that can be improved by a healthy weight.					
	4. I believe others judge me unfairly based on my weight					
	5. A healthy weight would help me achieve my personal/professional goals					
	6. A healthy weight would improve my depression, anxiety, or stress					
	External cues					
	7. A physician/nurse/dietitian advised me to be at a healthy weight					
	8. A loved one developed a serious health problem from being overweight or obese					
	9. A family member or close friend advised me to be at a healthy weight					
	10. I was presented information about the health risks of being overweight/obese in a college course					
	11. I read on a radio, television or social media website about the health risks of being overweight/obese					

	12. I saw an ad for a product or service that claimed to help me be at a healthy weight					

**Perceived self-efficacy in dieting**	Completely disagree	Disagree	Undecided	Agree	Completely agree
Habits and preferences subscale					
1. I can eat three meals regularly.					
2. I can eat meals in moderate amounts.					
3. I can eat fresh food rather than processed one					
4. I can refrain to eat sweets like candy and cookie.					
5. I can refrain to eat fatty food like fried food.					
6. I can refrain to drink carbonated drink like cola					
7. I can eat various foods to avoid unbalance in diet.					
8. I can refrain to eat just before going to bed.					
9. I can eat slowly even when hungry					
10. I can stop to eat before filling stomach even the food is delicious.					
11. I can get up early to eat breakfast.					
12. I can refrain to watch TV or read book when I eat.					
13. I can refuse to eat when my family members or friends offer foods.					
Emotional/mental health subscale					
14. I can refrain to eat when I am bored.					
15. I can refrain to eat when I am hungry.					
16. I can refrain to eat when I am angry.					
17. I can refrain to eat when I am depressed.					
18. I can refrain to eat when I am anxious and excited.					

**Perceived self-efficacy in exercise**	Completely disagree	Disagree	Undecided	Agree	Completely agree
1. I can walk for distance as far as 15 minutes walk.					
2. I can exercise until getting short of breath.					
3. I can exercise in cold weather.					
4. I can exercise in hot weather.					
5. I can exercise with friends after university.					
6. I can exercise instead of watching TV in leisure time.					
7. I can use stairs instead of elevators.					

**Behavioral intention of weight management**	Completely disagree	Disagree	Undecided	Agree	Completely agree
Diet therapy subscale					
1. I intend to control diet to reduce weight within six months.					
2. I intend to visit dietitian to reduce weight within six months.					
3. I intend to attend obese class if my college opens it.					
Exercise therapy subscale					
4. I intend to exercise by myself regularly to reduce weight within six months.					
5. I intend to attend sport centre or class to reduce weight within six months.					
